# Strategies in Developing Immunotherapy for Pancreatic Cancer: Recognizing and Correcting Multiple Immune “Defects” in the Tumor Microenvironment

**DOI:** 10.3390/jcm8091472

**Published:** 2019-09-16

**Authors:** Sireesha Upadhrasta, Lei Zheng

**Affiliations:** 1 The Sydney Kimmel Comprehensive Cancer Center, Johns Hopkins University School of Medicine, Baltimore, MD 21287, USA; 2 The Pancreatic Cancer Precision Medicine Center of Excellence Program, Johns Hopkins University School of Medicine, Baltimore, MD 21287, USA; 3 Department of Oncology, Johns Hopkins University School of Medicine, Baltimore, MD 21287, USA; 4 Department of Internal Medicine, Saint Agnes Hospital, Baltimore, MD 21229, USA; 5 Department of Surgery, Johns Hopkins University School of Medicine, Baltimore, MD 21287, USA

**Keywords:** pancreatic ductal adenocarcinoma, immune defect, immune checkpoint, myeloid cells, tumor microenvironment, stroma

## Abstract

With the advent of cancer immunotherapies, significant advances have been made in the treatment of many tumor types including melanoma, lung cancer, squamous cell carcinoma of the head and neck, renal cell carcinoma, bladder cancer, etc. However, similar success has not been observed with the treatment of pancreatic cancer and all other immunogenic “cold” tumors. This prompts the need for a better understanding of the complexity of the cold tumor microenvironment (TME) of pancreatic cancer and what are truly the “defects” in the TME making the cancer unresponsive to immune checkpoint inhibitors. Here we discuss four major immune defects that can be recognized in pancreatic cancer, including lack of high-quality effector intratumoral T cells, heterogeneous dense stroma as a barrier to effector immune cells infiltrating into the tumor, immunosuppressive tumor microenvironment, and failure of the T cells to accomplish tumor elimination. We also discuss potential strategies for pancreatic cancer treatment that work by correcting these immune defects.

## 1. Introduction

**Figure 1:**
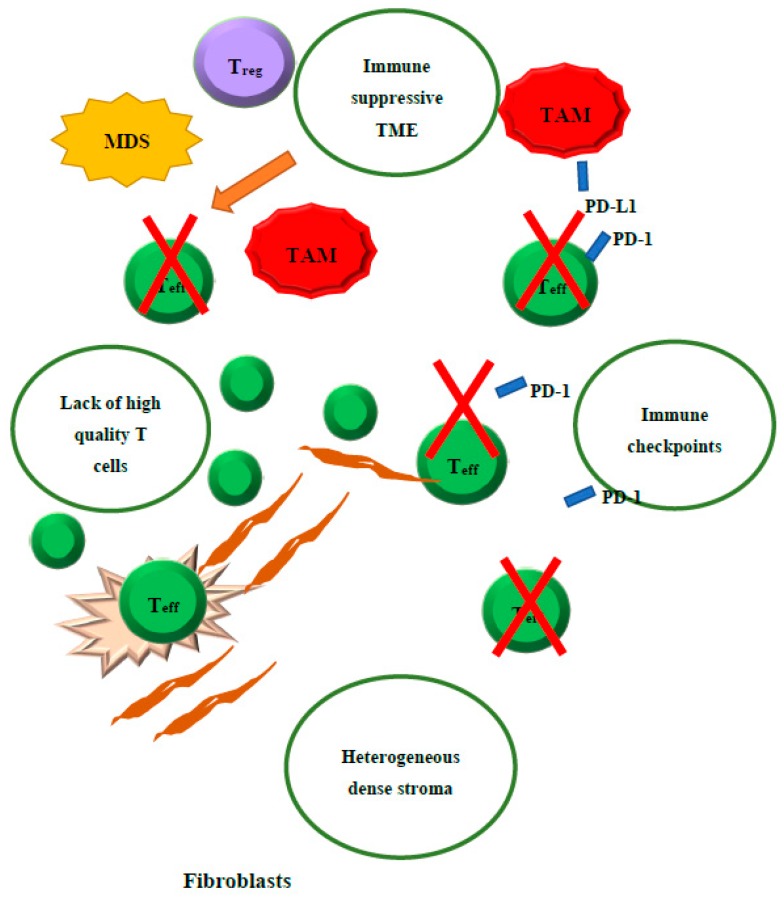
Illustration of the four main immune “defects” in pancreatic cancer.

Despite recent breakthroughs in cancer therapy, pancreatic ductal adenocarcinoma (PDAC), the primary cancer of the pancreas, continues to have a dismal outlook. In 2019 an estimated 56,770 new cases of pancreatic cancer were diagnosed in the USA (29,940 in males and 26,830 in females), of which 45,750 people died of the disease (23,800 deaths in males and 21,950 deaths in females), representing the third most common cause of cancer death [1]. The poor outcome in PDACs have been attributed to late diagnosis, early metastatic dissemination, and ineffective systemic therapies [2]. While immunotherapy, particularly immune checkpoint inhibitors, has become a breakthrough treatment modality for many different types of solid tumors, one wonders what accounts for the resistance of PDAC to immunotherapy. Accumulated evidence has suggested that PDAC is impaired with multiple “immune defects” including a lack of high-quality effector cells, barriers to effector cell infiltration due to heterogeneous dense stroma, an immunosuppressive tumor microenvironment (TME), and immune checkpoint signaling (Figure 1). Such “defects” are not immunodeficiencies that result in a lack of defense against infectious agents, but are the reasons for the failure to eliminate tumor cells by immune mechanisms. Malignant diseases that are sensitive to immune checkpoint inhibitors usually have only a single immune defect in the elimination step. Malignant diseases such as PDACs that are resistant to immune checkpoint inhibitors often have multiple immune defects. Thus, combination immunotherapies may not be successful unless they aim at correcting all these immune defects. Current immunotherapy combination strategies target one or two immune defects, but do not aim to correct all the immune defects (Table 1) [3].

## 2. Lack of High-Quality Effector T Cells in PDAC Tumors

PDAC is known for its low immunogenicity, which is now assumed to be related to its low mutation burden. As mutated proteins are the main source of neoantigens, low mutation burden, as demonstrated in the vast majority of microsatellite stable PDACs, would result in a low neoantigen burden and subsequently explains the lack of tumor-infiltrating effector T cells [4]. The number of CD8+ cells, which are correlated with good clinical response to immunotherapy, is significantly lower in the TME of “nonimmunogenic” cancers such as PDAC compared to “immunogenic” cancers such as melanoma [5]. Our study [6] found that vaccine therapy was able to induce the infiltration of CD8+ T cells in patients who received cancer vaccine treatment as a neo-adjuvant therapy. However, the number of CD8+ T cells in PDAC following the vaccine therapy was no longer predictive of longer survival. Instead, the number of granzyme B+ CD8+ cells was correlated with longer survival. This suggested that the quality of T cells may be more important than the number of T cells for the antitumor immune response. Nevertheless, it was noted that granzyme B+ cells were not adequately present in the vicinity of tumor cells [6]. The study by Balachnadran et al. [7] further demonstrated that granzyme B+ T cells, representing a group of T cells with high quality, are associated with long-term survivors, who comprise a very small subgroup among PDAC patients. This study also showed the presence of neoantigens in PDAC of patients with long-term survival. Although the total neoantigen burden is not correlated with survival, the presence of high-quality neoantigens that differ markedly from self-peptides does correlate with survival, further highlighting the importance of tumor-specific CD8 T cells in the long-term control of PDAC. Nevertheless, the majority of PDACs lack high-quality T cells.

## 3. Heterogeneous Dense Stroma

The stroma of PDAC is heterogeneous and composed of various cellular and extracellular components including fibroblasts, myofibroblasts, pancreatic stellate cells, immune cells, blood vessels, the extracellular matrix, and soluble proteins such as cytokines and growth factors [8]. These cellular components have the propensity to promote tumor progression and metastasis [9]. The dense stroma of PDAC also results in a high hydrostatic pressure within the vessels of the tumor and limits the trafficking of lymphocytes in mouse models of PDAC. It is intriguing to test whether targeting the stroma may facilitate the recruitment of lymphocytes in human PDACs [10]. Multiple mechanisms have been studied to target the dense stroma components such as hyaluronan. Hyaluronan (HA) is a large linear polysaccharide and one of the major components of the extracellular matrix in many solid tumors [11]. It has the physical property of binding to water avidly, thus creating an immobile gel-fluid phase that can cause vascular collapse [11]. Accumulation of HA in PDAC is associated with increased disease aggressiveness and decreased overall survival (OS) [12]. A number of preclinical studies in mouse models of PDAC have demonstrated the effectiveness of pegvorhyaluronidase alfa (PEGPH20), a pegylated recombinant human hyaluronidase, in improving vascular perfusion and reducing the barrier so small molecule anticancer therapeutics can access cancer cells [11]. For example, the addition of PEGPH20 to gemcitabine resulted in enhanced delivery of gemcitabine to the tumor and an 83% increase in survival, as well as a decrease in metastatic burden in mouse PDAC models [13]. Both phase Ib and phase II clinical trial trials of testing PEGPH20 with either gemcitabine or the combination of gemcitabine and nab-paclitaxel have led to significantly improved progression-free survival (PFS) in patients with HA-high metastatic PDAC [14,15]. However, another phase 2 study showed that the FOLFIRINOX chemotherapy in combination with PEGPH20 was inferior to FOLFIRINOX alone for metastatic PDAC patients [16]. The difference in the results of two PEGPH20-based clinical trials may be attributed to the different schedules of PEGPH20 used in different clinical trials. Nevertheless, the potential role of PEGPH20 in overcoming the barrier to intratumoral trafficking of immune cells is still intriguing.

The hedgehog (Hh) signaling pathway was implicated as playing a role in regulating the dense stroma of PDAC [17]. The Hh signaling cascade, along with its ligand sonic hedgehog (Shh) produced by tumor cells, leads to the activation of the Gli family of receptors, which, in turn, releases the repression on Smoothened1 (Smo) in stromal fibroblasts and leads to the proliferation of stroma fibroblasts [18]. Inhibiting the Hh pathway by small molecule inhibitors of Smo resulted in a less dense stroma, with a better penetration of chemotherapy into the stroma, leading to tumor shrinkage in mouse models with PDAC [19]. However, clinical trials failed to demonstrate the benefit of combining Hh inhibitors with chemotherapy in treating metastatic PDAC [20]. Subsequent studies in mouse PDAC models further demonstrated that genetically targeting the Hh signaling would lead to more rapid development of metastases and worsening survival of the mice [21,22]. These results suggest that the stroma functions by restricting cancer initiation and metastasis formation in the pancreas and the distant organs, respectively. However, it remains a puzzle how and when the stroma acquires the function of cancer promotion. It is possible that the function of the stroma is reprogrammed from a cancer-restrictive one to a cancer-permissive one during the course of the cancer development. Our group’s published study also suggested that there is a spatial heterogeneity of the stroma function. We observed that there is a heterogeneous distribution of Hh signaling intratumorally and intertumorally in PDACs [23]. Hh inhibition would lead to compensation by the hepatic growth factor (HGF) signaling that distributes in a stroma region different from where the Hh signaling distributes. As anticipated, the combination therapies that inhibit both Hh and HGF signaling demonstrated significantly stronger anti-PDAC activity [24].

Cancer-associated fibroblasts (CAFs) are the cellular component of the PDAC stroma. They are known to selectively secrete a serine protease, fibroblast activation protein (FAP) [25]. Several studies have implicated FAP in tumor growth and progression [26,27]. Lee et al. [28] tested the hypothesis that FAP enzymatic activity modifies the extracellular matrix and thus promotes the formation of permissive TME and tumor invasion in PDAC. The study reported that FAP-associated tumor invasiveness is mediated by β1-integrin and focal adhesion kinase (FAK), and that blocking FAP activity can lead to reduced invasiveness of PDAC. Feig et al. [29] linked the high expression of FAP by CAF in the tumor stroma to the resistance to anti-PD-1/PD-L1 immune checkpoint inhibitor therapy. FAK inhibition has also been evaluated as a synergistic modality along with immunotherapy and traditional chemotherapy. Transgenic PDAC mice treated with FAK inhibition in combination with gemcitabine or anti-PD1 antibody had a 2.5-fold increase in median survival time compared to gemcitabine or anti-PD1 antibody alone [30]. Chemokine receptor 4 (CXCR4) has been implicated in mediating local immunosuppressive activity of these FAP^+^ CAFs that express CXCL12, the ligand of CXCR4 [10,31]. Thus, inhibition of CXCR4 in combination with anti-PD-1/PD-L1 therapy could possibly improve the immune response in PDAC. Accumulated evidence has demonstrated that CAFs are educated by neoplastic cells at the epigenetic/transcriptional level and phenotypically reprogrammed. This phenotypic reprogramming of CAFs may further protect tumors from immune surveillance [32,33,34].

## 4. Immunosuppressive Tumor Microenvironment

Another mechanism underlying the poor responsiveness of PDAC to immune checkpoint inhibitor is apparently the dominance of immunosuppressive cells in the TME. Clark et al. [35] demonstrated in a genetically engineered mouse model of PDAC that immunosuppressive cells such as tumor-associated macrophages (TAMs) tumor-associated fibroblasts, myeloid-derived suppressive cells (MDSCs), and T regulatory cells (Tregs) form a major component of the TME even at early stages of premalignant lesions, namely PanINs, and are increased during the course of tumor progression from premalignant lesions to invasive and metastatic PDAC. Although the M1 type of TAMs is anticipated to be tumor-suppressive, due to tumor-derived factors such as hypoxia in the TME, TAMs in the PDACs are skewed toward M2 macrophages, which are procancerous [36]. Studies of the TME in human PDACs revealed that effector T cells in a subset of advanced PDACs showed no evidence of activation and were associated with the presence of intratumoral MDSCs [37,38]. Targeted depletion of a single myeloid subset, the granulocytic MDSC, showed an improved T cell response [39]. Treg cells are present at higher numbers both in the peripheral blood and in the TME of patients with invasive PDAC compared to healthy controls [40]. Owing to their immunosuppressive nature, Tregs likely contribute to the poor immune response of PDAC. Thus, MDSC, TAM, and Treg are important targets for cancer immunotherapy for PDACs.

Saung et al. [41] examined the effect of the therapeutic blockade of CSF-1R, the receptor of CSF-1, which plays an important role in myeloid cell differentiation. The study demonstrated that anti-CSF-1R blockade antibody, in combination with anti-PD-1 antibody, can enhance the expression of costimulatory molecules including OX40 and CD137 on otherwise exhausted PD-1+ T cells, suggesting that targeting myeloid cells through the anti-CSF-1R antibody can enhance the activation and proliferation of reinvigorated T cells. Beatty et al. [42] examined the CD40-mediated tumor regression in a genetically engineered KPC mouse model of PDAC. The CD40 agonist antibody was shown to activate macrophages, which lead to tumor regression and effector T cell infiltration. CSF-1R, CD40, and CXCL12/CXCR4 have become therapeutic targets on myeloid cells and their targeted therapies and have been tested in clinical trials. In a recent clinical trial of gemcitabine/abraxane plus anti-CD40 agonist antibody, with or without anti-PD-1 blockade antibody, it achieved an objective response rate of more than 50% in patients with untreated, metastatic PDACs (2019 AACR Abstract CT004; NCT02482168). Mechanistically, the anti-CD40 agonist antibody may facilitate T cell activation; the combination of chemotherapy and anti-CD40 agonist antibody may activate the macrophages to destruct the stroma barrier for effector T cell infiltration and serve as a vaccine therapy, as described above, to convert cold TMEs into hot ones [42].

In addition to those immune cells that harbor the immunosuppressive function, as discussed above, stromal fibroblasts are also immunosuppressive cells. The depletion of FAP-expressing cells, predominantly stromal fibroblasts, has been shown to allow immunological control of growth in mouse models of PDAC [31]. In addition, PDAC cells produce immunosuppressive cytokines such as TGF-β [43] and express surface molecules or circulating molecules such as FasL, PD-L1, and BTLA that mediate immune suppression [44,45,46].

## 5. Immune Checkpoint

An immune defect in tumor elimination exists universally in all types of solid tumors, including PDACs. Attempts to correct the above immune “defects” to improve antitumor immunity would induce immune checkpoint signaling. PD-L1, the ligand of PD-1, is upregulated in tumor cells in response to pro-inflammatory cytokines such as IFNγ [47,48]. PDACs that have been treated with vaccine therapy, chemotherapy, and/or radiation therapy showed an increased expression of PD-L1 on tumor epithelia [49]. However, enhanced PD-L1 expression does not necessarily sensitize PDAC to anti-PD-1/PD-L1 antibody therapies. Vaccine therapy induced the expression of PD-L1 on tumor cells and myeloid cells in PDAC and also induced the infiltration of PD-1+ effector T cells in the TME of PDAC [6]. By contrast, chemotherapy and radiation therapy do not induce the infiltration of effector T cells into PDACs in association with the induction of the expression of PD-L1 (unpublished results). Therefore, a T cell-generating agent may be necessary for the combination of chemotherapy or radiation therapy and immune checkpoint inhibitor to demonstrate the effectiveness in PDAC patients.

Vaccine therapy also upregulates the expression of indoleamine 2,3-dioxygenase (IDO), an enzyme that disrupts the tryptophan metabolism in T cells and thus impairs T cell functions. Higher IDO expression in PDAC correlates with poorer survival in patients. Similar to PD-L1 expression, vaccine therapy induced IDO expression in both tumor cells and myeloid cells in PDACs, also as a result of the effector T cell infiltration. On the other hand, although chemotherapy and radiation therapy also induce the expression of IDO, these therapeutic modalities do not lead to increased effector T cell infiltration in PDAC [23]. Inhibition of IDO can be a potentially effective strategy to enhance the antitumor immune response in PDAC patients [23], if combined with an agent that also induces effector T cell infiltration. However, after PDAC is converted from a noninflamed tumor to an inflamed one like melanoma as the result of an agent that induces effector T cell infiltration, further combining the anti-PD-1/PD-L1 antibody treatment with IDO inhibitor with the anti-PD-1/PD-L1 antibody treatment would not result in additional antitumor activity in the preclinical model of PDAC. It would be intriguing to combine IDO inhibitors with immunotherapeutics such as vaccine therapy (NCT03006302) and myeloid cell-targeting agents that are aimed at correcting other immune defects.

## 6. Strategies for Developing Immunotherapy for Pancreatic Cancer

**Figure 2:**
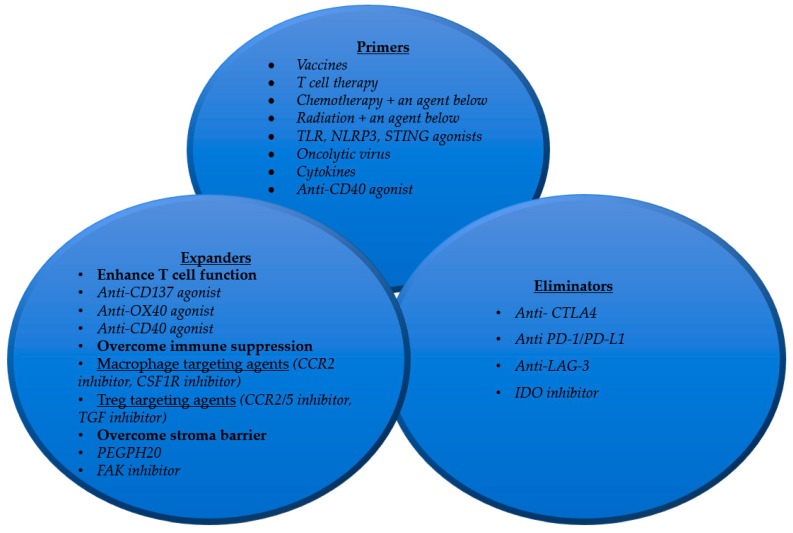
Strategies for developing combination immunotherapy to correct the immune “defects” in pancreatic cancer.

It is important to recognize that patients with pancreatic cancer may demonstrate a combination of more than one of the immune defects discussed above. More challenging is that immune defects are subject to change through the treatment course. Often, a new immune defect is unmasked after treating the immune defect that was initially recognized. Therefore, a combinational immunotherapy strategy that can correct all four immune defects is needed for essentially all pancreatic cancer patients (Figure 2). To correct for the lack of high-quality effector T cells, a “primer” treatment would need to be developed. Such a treatment could be a cancer vaccine or a T cell therapy. Conventional therapies such as chemotherapy and radiation may not be an appropriate primer as they by themselves do not generate T cells in the TME. Chemotherapy and/or radiation therapy may need to be combined with cytokines, TLR ligands, NLRP ligands, and STING agonists [50,51] to achieve the effect of in situ vaccination. By contrast, a cancer vaccine generates T cells peripherally, which may not infiltrate efficiently into the tumors. Direct T cell therapy may encounter the same challenge. Moreover, T cells generated by a primer would be quickly exhausted if a checkpoint inhibitor treatment such as anti-PD-1 antibodies is not combined. Therefore, treatment with immune checkpoint inhibitors is still essential for the immune-based tumor elimination step. Immune checkpoint inhibitors permit the effector T cells to eliminate tumor cells and thus function as an “eliminator.” However, even if the treatments with primer and eliminator are given, the antitumor function of T cells is still suppressed by nonpermissive and immunosuppressive TME. Thus, “expanders” would need to be developed to make the TME more permissive for T cell infiltration, to modulate the immunosuppressive components in the TME, and to maintain a highly proliferative, active, memory, and cytotoxic status of effector cells. To these ends, more effective and specific agents that modulate the immunosuppressive components are in demand. Alternatively, T cell activation agents would be needed to proactively expand antitumor effector T cells. Agents that target the stroma components not only can remove the barrier to T cell infiltration, but may also reverse the immunosuppressive functions of the stroma [52]. A stroma targeting agent may be an indispensable component of an expander treatment for tumors with dense stroma. The above combination therapy in the clinical trial with chemotherapy, anti-CD40 agonist antibody, and anti-PD-1 blockade antibody is an example of targeting multiple immune defects in PDACs; such a combination therapy strategy may be potentially superior to those listed in Table 1.

It is important to note that each pancreatic cancer patient is different and hence patient characteristics such as genetic variability, age, and indications for treatment should be taken into consideration when choosing the proper combinational immunotherapy for each individual patient. Tumor genetics and the patients’ genetic variability may hold the key to choosing the right immunotherapy combination for a patient at the right time [52].

## Figures and Tables

**Table 1:** Selected examples of completed and ongoing combination immunotherapy clinical trials/studies.

Target Resistance Mechanism	Combination	Agents	Tumor Type	Study/Results
Priming tumor microenvironment	Vaccine and Checkpoint inhibitor	Ipiliimumab+/− GVAX	Metastatic PDAC	Objective responses were observed in 20% in combination arm, none of the patients responded to single agent anti-CTLA4 therapy. NCT00836407
		Cyclophosamide/ GVAX+/− Nivolumab+/− Urelumab	Resectable PDAC	Ongoing NCT02451982
	Checkpoint Inhibitor (CTLA-4, PD-1) + Radiation	SBRT, Tremelimumab, Durvalumab	Metastatic PDAC	Ongoing NCT02311361
	Radiation Therapy + Checkpoint inhibitor + Vaccine	GVAX SBRT Cyclophosamide, pembrolizumab	Locally Advanced PDAC	Ongoing NCT02648282
Modulating Tumor Microenvironment	CSF-1R Inhibitor Checkpoint Inhibitor (PD-1)	Nivolumab Cabiralizumab Chemotherapy	Metastatic PDAC	Ongoing NCT03336216
	FAK inhibitor + Chemotherapy + checkpoint Inhibitor	Neoadjuvant and Adjuvant Chemotherapy Pembrolizumab+/− Defactinib (FAK inhibitor)	Resectable PDAC	Ongoing NCT03727880
	CXCR2+ Checkpoint Inhibitor+ Chemotherapy	AZD5069 Duvalumab Chemotherapy	Metastatic PDAC	Completed NCT02583477
	Recombinant hyaluronidase + Checkpoint inhibitor (Anti-PD-L1)+ Chemotherapy	PEGPH20 Atezolizumab Chemotherapy	Metastatic PDAC	NCT03267940
